# Influence of Light-Emitting Diode-Derived Blue Light Overexposure on Rat Ocular Surface

**DOI:** 10.1155/2023/1097704

**Published:** 2023-01-10

**Authors:** Li Nan, Yifan Zhang, Hui Song, Yan Ye, Zhixin Jiang, Shujun Zhao

**Affiliations:** ^1^Tianjin Eye Hospital, Tianjin Key Lab of Ophthalmology and Visual Science, Nankai University Affiliated Eye Hospital, Clinical College of Ophthalmology Tianjin Medical University Tianjin Eye Institute, Tianjin, China; ^2^NHC Key Laboratory of Hormones and Development, Tianjin Key Laboratory of Metabolic Diseases, Chu Hsien-I Memorial Hospital & Tianjin Institute of Endocrinology, Tianjin Medical University, Tianjin, China

## Abstract

We aim to investigate the effect of overexposure to blue light on the rat ocular surface and explore the potential mechanisms. 450 nm light-emitting diode (LED) derived light at 1000 lux was used to irradiate SD rats, 12 hours a day, for consecutive 28 days. Rats in the control group were exposed to 400 lux white light at the same time (in an indoor environment). Tear film breakup time (TBUT), tear volume, and corneal fluorescein staining scores were used to measure the changes to the ocular surface. Expressions of nuclear factor-*κ*B (NF-*κ*B), inhibitor-*κ*B (I-*κ*B), interleukin-6 (IL-6), and tumor necrosis factor-*α* (TNF-*α*) were measured by real-time PCR, and the activation of the NF-*κ*B pathway was detected by Western blotting, respectively. Cornea ultrastructure was examined by TEM and optical microscope on day 28. Pyrrolidine dithiocarbamate (PDTC), an inhibitor of NF-*κ*B signaling pathway, was used to measure the inhibition of blue light injury. The above indexes were detected again when compared with the solvent-treated group. On day 28, compared with day 0, the TBUT of the blue light group was significantly shorter, and the score was significantly higher. The amount of tear secretion changed slightly with time. HE and PAS staining revealed significantly decreased corneal epithelial cell layers and goblet cells after 28-day irradiation of blue light. Disarranged stromal cells, vacuoles in the basal nuclei, and decreased desmosomes were also found in the blue light group. Significantly increased levels of NF-*κ*B, IL-6, TNF-*α*, and the ratio of phosphorylated NF-*κ*B p65 (pNF-*κ*B p65) to total NF-*κ*B p65 implied blue light-induced damage and pathway activation. In addition, PDTC significantly reduced the phosphorylation of NF-*κ*B activated in blue light-treated corneas and alleviated the ocular surface changes caused by blue light. Finally, our results demonstrated that long-term blue light exposure in rats could cause ocular surface changes and manifest as dry eye. Inflammation and activation of the NF-*κ*B pathway may play a role in the pathogenesis.

## 1. Background

Light pollution is becoming an inevitable problem with the wide use of artificial light in daily life. Light-emitting diode (LED) is a common light source, which is increasing in computers, smartphones, and room illumination [1]. Because of its special spectrum, more and more people began to pay attention to its biosecurity [2]. In the visual spectrum of light, the wavelength between 415 and 455 nm blue light is closely related to eye damage [3]. It is often emphasized that LED-based light sources are different from traditional lamps in that they contain higher proportions of blue wavelength light and are thus more likely to cause problems such as blue-light hazards [4].

The negative effect of blue light LED on the retina has been extensively studied [5, 6]. Indeed, the accumulation of blue light can also disturb the balance of the eye's surface environment [7, 8]. The ocular surface is the first barrier through which light enters the eye, and it is more susceptible to environmental changes [9, 10]. Researchers have indicated that blue light with short wavelengths (410–480 nm) could induce oxidative damage to the corneal epithelium [11] and may be hazardous to corneal epithelial cells undergoing mitosis [12]. Blue LEDs could also aggravate dry eye clinical parameters in mice compared with red or green light [7]. However, little attention was paid to the influence of blue light toxicity on the ocular surface and its mechanism in vivo. The relationship between blue light exposure and dry eyes and the pathogenic mechanism are not well understood.

The NF-*κ*B pathway is a transcription factor pathway related to inflammation, apoptosis, and stress response, which has been proven to be involved in the pathogenesis of a variety of ocular diseases [13]. Overexposure of blue light would cause RPE and photoreceptor damage [14], and the pathogenesis might be associated with NF-*κ*Β pathway activation [15]. It has also been shown that PDTC can relieve ocular surface symptoms in mice with allergic conjunctivitis by inhibiting downstream reactions of the pathway [16]. Thus, we inferred that the damage of blue light on the ocular surface and the symptoms of dry eye are related to the NF-*κ*B pathway. At the same time, ocular surface light damage may be relieved by PDTC. So, we aimed to investigate the effect of blue light overexposure on dry eye in a rat model and explored the potential mechanism between blue light irradiation and ocular injury, in order to find potential treatments.

## 2. Methods

### 2.1. Animals

Twenty-four six-week-old clean female SD rats were used in this research. Animals were bred and housed in our convention facility with a 12-h light/dark cycle (Nankai Hospital animal facilities). The facility temperature was maintained at 25 ± 2°C with the relative humidity of 40 ± 5%. No abnormality was observed in the anterior segment when examined with a slit lamp microscope before the research. The animal use protocol listed below has been approved by the Animal Ethical and Welfare Committee (Chinese Academy of Medical Sciences, Institute of Radiation Medicine).

### 2.2. Study Groups and Treatments

All rats were randomly divided into four groups: (a) normal control group (control group, *n* = 6); (b) blue light irradiation group (blue light group, *n* = 6); (c) blue light irradiation with the saline treatment group (B + S group, *n* = 6); (d) blue light irradiation with pyrrolidine dithiocarbamate (PDTC) treatment group (B + P group, *n* = 6).

### 2.3. Animal Experimental Procedure

The control group was untreated for baseline comparison. Rats in the other three groups were exposed to 1000 lux blue light 12 hours per day (from 7 : 00–19 : 00) for consecutive 28 days. 400 lux domestic white light was used to irradiate rats in the control group at the same time. For effective exposure, animals in the treatment groups were separated in adjustable cages in the blue light facility (Jingyi Optoelectronics Technology Co. Ltd., Guangzhou, China), where the LED panel was placed 5 cm above the rats' heads. The LED lights with a wavelength of 450 nm were used in this study, and the luminance was measured by a digital luxmeter (Taiwan TES Electronics Industry Co., Ltd., TES1332A) every time before the irradiation.

For NF-*κ*B inhibition, the NF-*κ*B activation inhibitor PDTC was used in this research. PDTC was diluted in saline to the concentration as described in the previous study [17]. During the irradiation, PDTC solution was dosed to both eyes three times a day (7 : 00; 12 : 00; 17 : 00), every time 5 uL of 0.1 mM by a pipette for 28 consecutive days. In the B + S group, rats were treated with a similar volume of saline at the same time.

The clinical parameter measurements, including tear breakup time (TBUT), tear volume, and corneal staining scores, were taken on day 0, day 14, and day 28. All rats were euthanized on day 28 by cervical dislocation. The right eyeballs were obtained for histological observation, while the cornea and conjunctiva of the left eye were harvested for real-time PCR and western blot analysis.

### 2.4. Dry Eye-Related Clinical Observation

Tear volume, fluorescein sodium staining, and TBUT were measured as previously described [18, 19]. In order to avoid the influence of anesthesia on the amount of tear secretion and the stability of the tear film, all operations were performed in a nonanesthetic state.

### 2.5. Tear Volume

The phenol red thread (Tianjin Jingming New Technology Development Co. Ltd., Tianjin, China) put into the lower eyelid conjunctival sac was used to measure aqueous tear secretion. After 20 s, the thread was withdrawn and the red thread length was measured. The measurements were repeated 3 times, and the average value was obtained.

### 2.6. Tear Breakup Time and Corneal Staining Score

A total of 5 *μ*L of 0.1% fluorescein sodium staining solution (Alcon Laboratories, Inc., USA) was applied to the lateral conjunctival sac of the mice with a micropipette. After three blinks, the corneal surface was carefully observed with low brightness cobalt blue light under the slip lamp microscope. The time was recorded when the first black hole in the tear film was found. The fluorescein punctate staining score was measured as it used to be [20]. Each cornea was divided into four quadrants that were scored individually. The intensity of corneal fluorescein staining was calculated using a 4-point scale: 0, absent; 1, superficial stippling micropunctate staining <30 spots; 2, punctate staining >30 spots but not diffuse; 3, severe diffuse staining but no positive plaque/patch; and 4, positive fluorescein plaque/patch. The scores of the four areas were summed to generate a final grade, ranging from 0 to 16. The measurements were repeated 3 times, and the average value was obtained.

### 2.7. Hematoxylin and Eosin (HE) and Periodic Acid–Schiff (PAS) Staining

The entire eyeballs fixed with 10% formalin were dehydrated by gradient ethanol, transparent in xylene, and embedded in paraffin. Tissue sections of 4-*μ*m thickness were stained with PAS (Sigma-Aldrich, St. Louis, MO, USA) and hematoxylin and eosin (Tianjin Chemical Reagent Factory, China). Three representative slices from the homologous positions of each sample were selected (three samples for each group). The numbers of epithelial cell layers and goblet cells from each sample were counted, and the average was calculated (cells/visual field, 200×). The ultrastructural changes of corneal epithelial cells were acquired and analysed by using the LeicaQ550CW image analysis system (Leica Microsystems GmbH, Wetzlar, Germany).

### 2.8. Transmission Electron Microscopy (TEM) Test

For the corneal morphology, the corneas of the right eye were harvested on day 28 and preserved in electron fixation solution for 24 hours at 4°C. Samples were processed as previously described [20]. In each group, we chose three samples for TEM examination. The morphological image was captured by a transmission electron microscope (HT7700, HITACHI, Tokyo, Japan).

### 2.9. Real-Time PCR

Total RNA was extracted from corneal tissues using a commercial reagent (TRIzol; Invitrogen, Carlsbad, CA). First strand cDNA was synthesized with random hexamers using reverse transcriptase (SuperScript III; Invitrogen), and quantitative real-time polymerase chain reaction (PCR) was performed using predesigned primers for interleukin-6 (IL-6), tumor necrosis factor-*α* (TNF-*α*), nuclear factor-*κ*B (NF-*κ*B), and inhibitor-*κ*B (I-*κ*B) with the SYBR Premix Ex Taq Kit. The GAPDH gene was used as the endogenous reference for each reaction. The results were analysed by the comparative threshold cycle (CT) method using commercial analysis software (LightCycler, version 3; Roche Diagnostics Corp., Indianapolis, IN).

### 2.10. Western Blot Analysis

The corneal tissues were lysed with cold RIPA buffer, and the total protein concentration was subjected to electrophoresis on an 10% SDS-PAGE gel with an equal amount. After being transferred to polyvinylidene fluoride (PVDF) membranes and blocked by 5% evaporated milk, proteins were incubated overnight with primary antibodies at 4°C. The following primary antibodies were used: phosphorylated nuclear factor-*κ*B p65 (pNF-*κ*B p65, 1 : 1000 Cell Signaling Technology, Danvers, MA, USA), NF-*κ*B p65 (1 : 1000, Cell Signaling Technology), TNF-*α* (1 : 1000, Abcam, Cambridge, UK), and *β*-actin (1 : 10000; Sigma, St. Louis, MO) was used as a loading control. HRP-conjugated goat antirabbit IgG (1 : 3000; Zhongshan Golden Bridge Biotechnology Co. Ltd., Beijing, China) was used as the secondary antibody. Densitometry of the Western blot bands was performed using the Image J software.

### 2.11. Statistical Analysis

Statistical data were analysed by the Statistical Package for the Social Sciences (SPSS) 22.0 program (SPSS Inc., Chicago, IL). The data of each test indicator in this study showed a normal distribution after the Shapiro–Wilk test, and the data in each group were uniformly tested by the Levene test. Results were presented as a mean ± standard deviation (SD). The overall differences in tear volume, TBUT, and corneal fluorescein staining among the groups were compared by two-way analysis of variance tests (two way-ANOVA). Bonferroni analysis was used for pairwise comparisons between groups. The independent sample *t*-test was used to compare cell numbers, cytokine, and protein value differences between two groups. *P* < 0.05 was considered statistically significant.

## 3. Results

### 3.1. Blue Light Aggravates Clinical Dry Eye Parameters in Rat Models

#### 3.1.1. Tear Breakup Time

**Figure 1:**
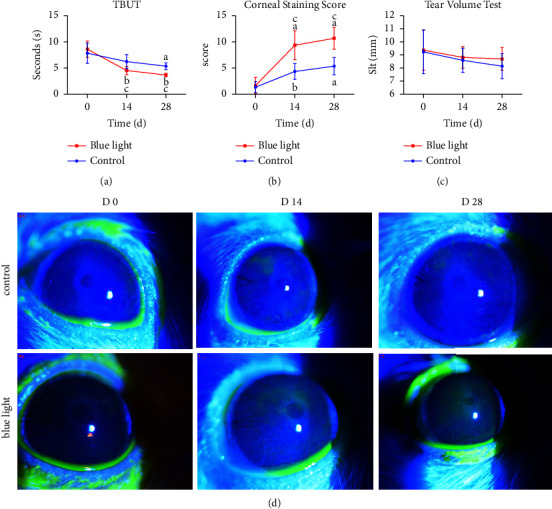
Examination of clinical dry eye parameters in normal and blue light-treated rat corneas. (a, b) Increased fluorescein staining and decreased TBUT were recorded in both groups. Compared with the control group, the blue light group showed significantly shorter TBUT and higher fluorescein sodium staining scores after blue light treatment. ^*a*^*P* < 0.01 compared with day 0; ^*b*^*P* < 0.001 compared with day 0; control vs. blue light: ^*c*^*P*  <  0.001. (c) Both groups showed no significant changes of tear secretion within and between groups. (d) Representative examples of fluorescein sodium staining on day 28 (right line, d) compared to day 14 (middle line, d) and day 0 (left line, d).

At day 0, there was no significant difference between the control and blue light-treated groups. The TBUT decreased significantly in the blue light group after 14 days irradiation (^*b*^*P* < 0.001, Figure 1(a)), while the value of the control group changed slightly. Although TBUTs of the two groups were shortened after 28 days compared with day 0 (^*a*^*P* < 0.01, ^*b*^*P* < 0.001, Figure 1(a)), the blue light-treated group showed significantly decreased TBUTs compared with that in the control group at day 14 and day 28 (^*c*^*P* < 0.001, Figure 1(a)).

#### 3.1.2. Corneal Staining Score

The mean staining score showed no statistically significant differences between the two groups at day 0 (*P* > 0.05). The corneal staining scores of different groups at different time points after the intervention were statistically significant (*F*_group_ = 34.133, *P*_group_ < 0.001; *F*_time_ = 43.233, *P*_time_ < 0.001). The blue light group showed significantly increased corneal staining scores on days 14 and 28 compared with that on day 0 (^*b*^*P* < 0.001, Figures 1(b), 1(d)). The score in the control group was also increased significantly on day 28 (^*a*^*P* < 0.001, Figure 1(b)). As for intergroup comparisons, the corneal staining score in the blue light group was significantly lower than that in the control group on day 14 and day 28 (^*b*^*P* < 0.001, Figure 1(b)).

#### 3.1.3. Tear Volume

Before the experiment, mean tear volume had no significant difference between the two groups (control: 9.22 ± 1.65 mm, blue light: 9.36 ± 1.53 mm, *P* > 0.05). In addition, no statistically significant differences were observed in tear volumes between the two groups during the experiment period (all *P* > 0.05, Figure 1(c)).

### 3.2. Morphological Analysis of Ocular Surface after Blue Light Overexposure

#### 3.2.1. HE Staining

**Figure 2:**
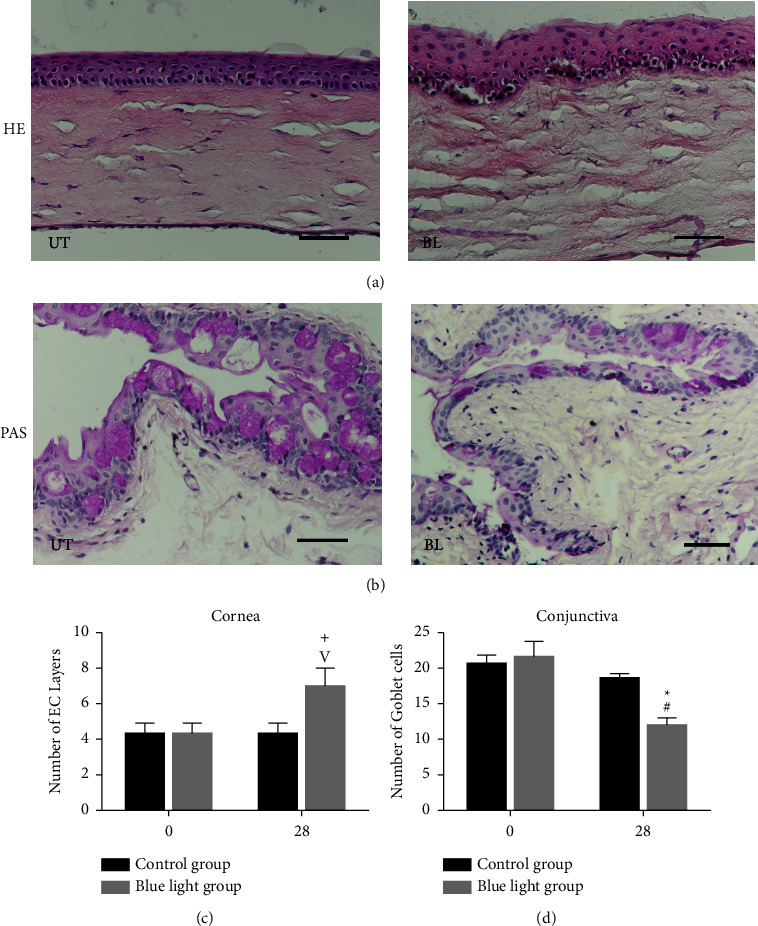
Histology analysis of corneal epithelium and goblet cells of the conjunctiva. (a, b) Representative images for PAS and HE staining are shown in the picture. In the blue light group, the arrangement of cells was disordered in each layer (right, a) with significantly increased epithelial cell layers (day 0 vs. day 28: ^+^*P* < 0.05, (c) after irradiation for 28 days. Furthermore, the number of goblet cells in the conjunctiva decreased significantly compared with the control group (c) and day 0 value (day 0 vs. day 28: ^#^*P* < 0.01, (d). Control group vs. the blue light group: ^*∗*^*P* < 0.01, ^*v*^*P* < 0.05. Scale bars: 50 *μ*m.

In the untreated group, the cornea was composed of four to five layers of corneal epithelial cells, and the stromal cells were arranged neatly (Figure 2(a), left column). After irradiation for 28 days, the corneal surface was not smooth (Figure 2(a), right column), and the number of corneal epithelial cells increased significantly with the disordered arrangement (^+^*P* < 0.01, Figure 2(c)).

#### 3.2.2. Goblet Cell Detection

Goblet cells in the conjunctiva were examined by PAS staining. There was no significant difference between the two groups on day 0. The number of PAS-positive cells was significantly decreased in the light-treated group, while the control group showed almost no change after 28 days (both *P* < 0.01, Figures 2(b) and 2(d)).

#### 3.2.3. TEM

**Figure 3:**
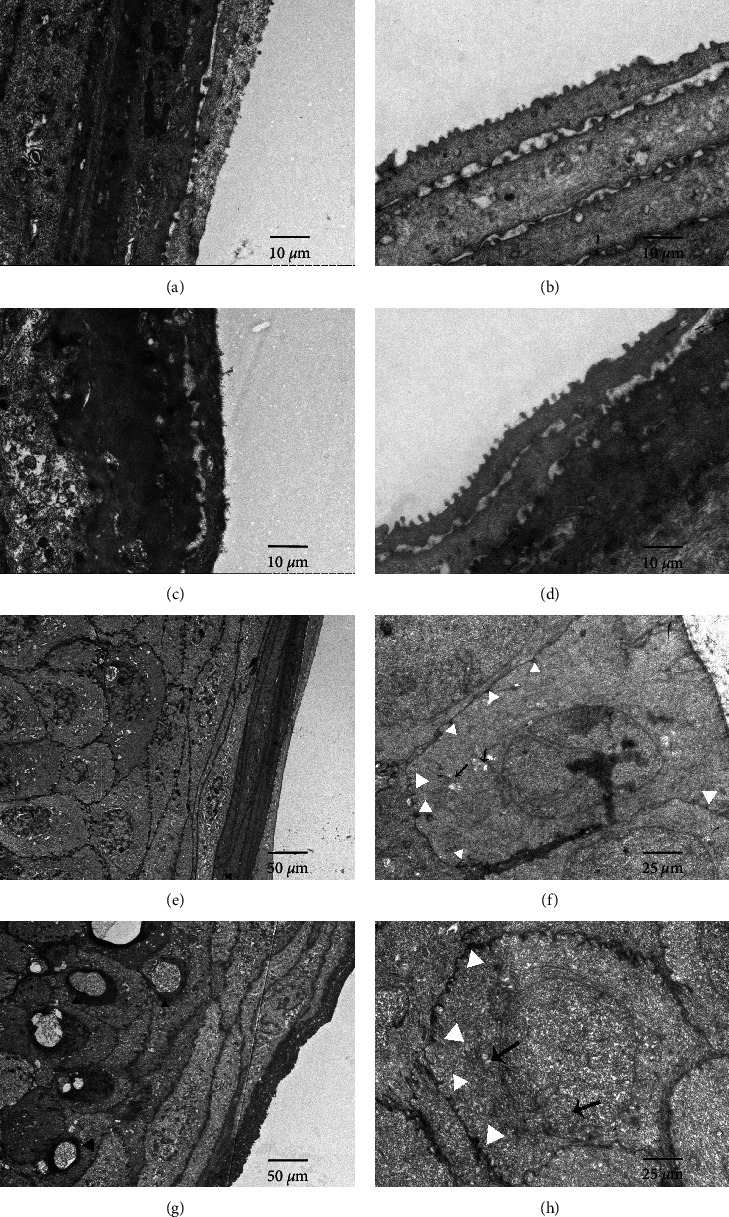
TEM in the cornea of rats. In the control group (a, b, e), TEM showed that the corneal flat epithelial cells were arranged in order. By contrast, decreased microvillus (d, g) and disordered stromal layer were observed (c, d) in the blue light group. Furthermore, vacuoles were found in the basal nuclei (g, black arrow head) compared to the unexposed group (e). Fewer desmosomes between adjacent cells were recorded after 4 weeks of blue light exposure (h, white arrow head) with mitochondria swollen, vacuolated, and cristae disappeared (h, black arrow) compared with the control group (f). Scales of magnification: (a–d): 4000x. (e, g): 700x. (f, h): 2000x.

Normal corneal epithelium cells have a smooth surface and neatly arranged microvilli (Figures 3(a), 3(b), and 3(e)) compared with blue light-induced samples (Figures 3(c) and 3(d)). There is no obvious damage to the corneal epithelium in the control group samples and no significant changes in intercellular connectivity were observed (Figure 3(f)). However, in a blue light sample, large vacuoles appeared in a few epithelial nuclei (Figure 3(g)), and the chromatin homogenization was changed. The nuclear envelope is dissolved and damaged. Compared with the control group, fewer desmosomes between adjacent cells were recorded after 4 weeks of blue light exposure (Figure 3(h)).

### 3.3. Blue Light Induces Dry Eye Syndrome via NF-*κ*B Activation

**Figure 4:**
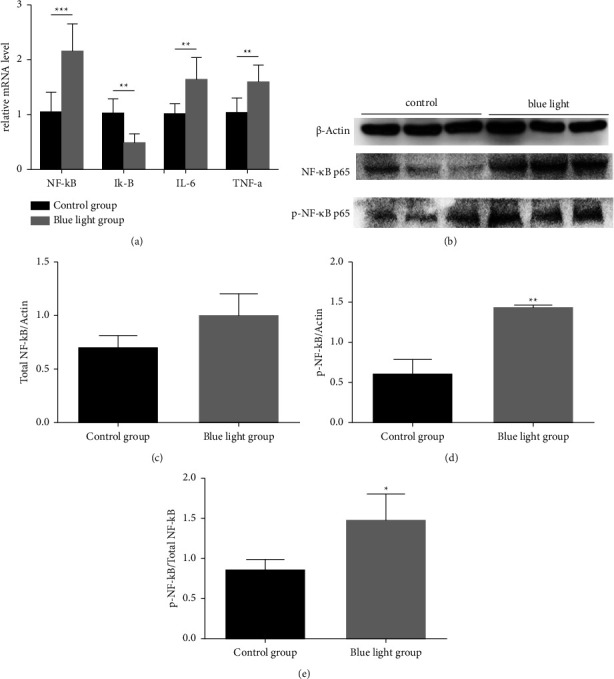
NF-*κ*B pathway-related proteins in the cornea after blue light irradiation. (a) The production of NF-*κ*B, I*κ*-B, IL-6, and TNF-*α* were evaluated by real-time PCR, with GAPDH as a loading control. After treatment for 28 days, the mRNA levels of NF-*κ*B, TNF-*α*, and IL-6 were upregulated and significantly higher than those in the control group (^*∗*^*P* < 0.05, ^*∗∗*^*P* < 0.01, ^*∗∗∗*^*P* < 0.001). (b–e) The effects of blue light on the activation of NF-*κ*B in corneas were evaluated by Western blot analysis using *β*-actin as a loading control. (b) After exposure for 28 days, the protein levels of total NF-*κ*B p65 and phosphorylated NF-*κ*B p65 were upregulated and higher than those in the control group. (c, d) The bands showed the statistical analysis of groups by density values. (e) The ratio of phosphorylated NF-*κ*B to total NF-*κ*B showed a significant difference compared with the control group. ^*∗*^*P* < 0.05, ^*∗∗*^*P* < 0.01.

To evaluate the mechanisms underlying blue light-induced dry eye, we analysed the activation of NF-*κ*B by Western blot analysis. Moreover, real-time PCR analysis was used to detect the expression of NF-*κ*B, TNF-*α*, and IL-1, and the specific cytokines in NF-*κ*B signaling pathway. The data revealed that TNF-*α*, IL-6, and NF-*κ*B levels were significantly higher in blue light-treated ocular surfaces compared to the control group samples (*P* < 0.01, *P* < 0.01, and *P* < 0.001, respectively). In addition, the level of I-*κ*B decreased significantly (*P* < 0.01, Figure 4(a)).

As for Western blot analysis, the protein level of total NF-*κ*B in the blue light-treated ocular surface was upregulated and higher than that in the control group (Figures 4(b) and 4(c)). In addition, the levels of phosphorylated NF-*κ*B were markedly increased in the ocular surface treated with blue light irradiation as well (*P* < 0.01, Figures 4(b) and 4(d)). Besides, blue light stimulation also significantly increased the ratio of phosphorylated NF-*κ*B p65 to total NF-*κ*B p65, compared with that in the control group (*P* < 0.05, Figure 4(e)).

### 3.4. PDTC Treatment Alleviated Blue Light-Induced Cornea Toxicity

#### 3.4.1. PDTC Treatment Relieves Dry Eye Symptoms

**Figure 5:**
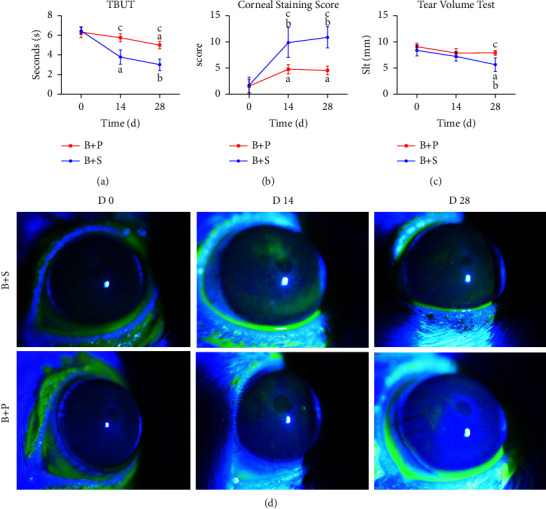
Clinical indicators of saline and the PDTC group after blue light stimulation. (a) The PDTC-treated group showed an increased TBUT compared to the saline-treated group (^*c*^*P* < 0.01). (b) Scores of fluorescence staining significantly increased with time (^*c*^*P* < 0.01, ^*b*^*P* < 0.05 compared with day 0 in the same group). PDTC showed a decreased score compared to the saline-treated group (^*c*^*P* < 0.01). (c) In the B + S group, tear secretion decreased in a time-dependent manner (day 0 vs. day 28: ^*a*^*P* < 0.01, day 28 vs. day 14: ^*b*^*P* < 0.05) while the index changed slightly in the B + P group. (d) Representative examples of fluorescein sodium staining.

Before the research, no statistically significant differences were observed in TBUT, corneal staining scores, and tear volume time between the B + S and B + P groups (all *P* > 0.05). Compared with the B + S group, PDTC was effective in prolonging TBUT (*F*_time_ = 94.492, *P*_(time) < 0.001; *F*_group_ = 15.223, *P*_(group) < 0.001). At day 14, TBUT in B + S was significantly reduced compared to the mean time in day 0, while the value decreased slightly in the B + P group (^*a*^*P* < 0.01 and *P* > 0.05, respectively). Although the TBUT of the B + P group decreased significantly at day 28, it was longer than that of the B + S group at any time point during the experiment (^*c*^*P* < 0.01, Figure 5(a)).

A significant difference in corneal staining was also found between the B + P group and the B + P group (*F*_time_ = 47.395, *P*_(time) < 0.001; *F*_group_ = 45.900, *P*_(group) < 0.001). At day 28, the B + S group showed an increased corneal staining score compared with the day 0 value (Figure 5(d)). The mean corneal staining score in the B + P group was significantly reduced compared with that in the B + S group at day 14 and day 28 (both *P* < 0.01, Figure 5(b)).

The tear volume of each group at day 14 showed no statistically significant difference when compared with that of each group at day 0 (*P* > 0.05, Figure 5(c)). On day 28, the amount of tear secretion decreased significantly compared with 14 days and 0 days (^*a*^*P* < 0.01 and ^*b*^*P* < 0.05, respectively, Figure 5(c)), while PDTC effectively retained tear secretion (B + S vs. B + P: ^*c*^*P* < 0.01).

These data may explain that PDTC treatment could protect the cornea from blue light-induced dry eye symptoms.

#### 3.4.2. PDTC Ameliorated the Structural Changes Induced by Blue Light

**Figure 6:**
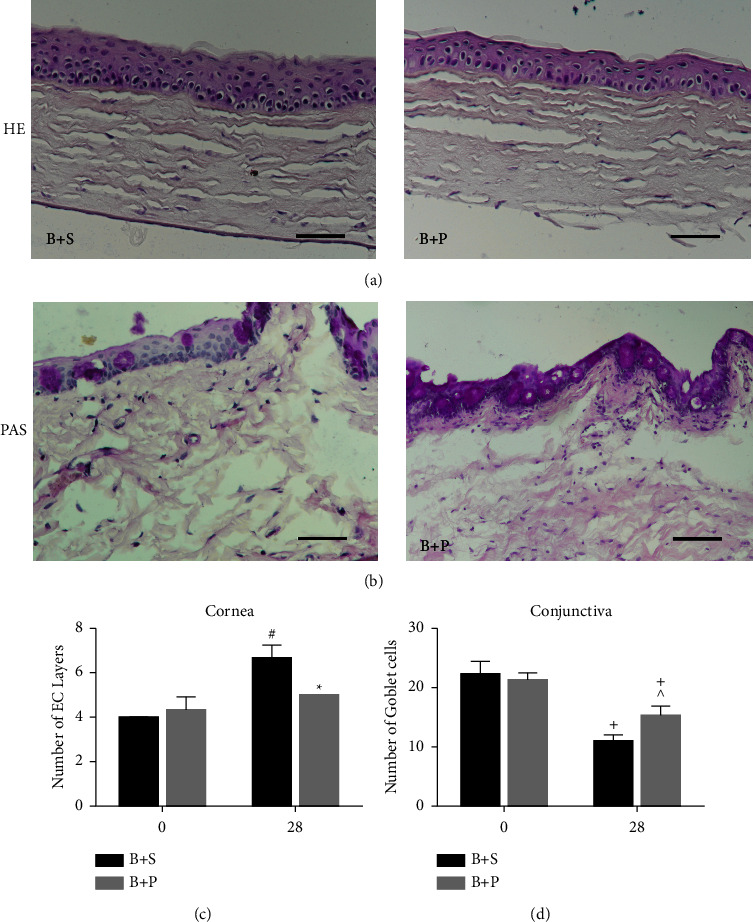
Alterations of corneal structures and goblet cells in the conjunctiva. (a, b) HE staining of corneal epithelium and goblet cells in the conjunctiva observed by PAS staining. Scale bars: 50 *μ*m. (c) Decreased epithelial corneal layers were recorded after PDTC treatment (B + P vs. B + S: ^*∗*^*P* < 0.01, day 28 vs. day 0: ^#^*P* < 0.05). (d) Number of goblet cells decreased over time (day 28 vs. day 0: ^+^*P* < 0.01). Compared to the B + S group, PAS staining showed markedly increased goblet cells in the B + P group (B + P vs. B + S: ^∧^*P* < 0.05).

As shown in Figure 6(a), the number of epithelial cells was significantly lower than that in the B + S group (B + P vs. B + S: ^*∗*^*P* < 0.01, Figure 6(c)). At the same time, the blue light treatment significantly decreased the density of goblet cells (^+^*P* < 0.01, Figure 6(d)) while PDTC rescued most of them (B + P vs. B + S: ^∧^*P* < 0.05, Figures 6(b) and 6(d)).

#### 3.4.3. Effect of PDTC on NF-*κ*B Activation

**Figure 7:**
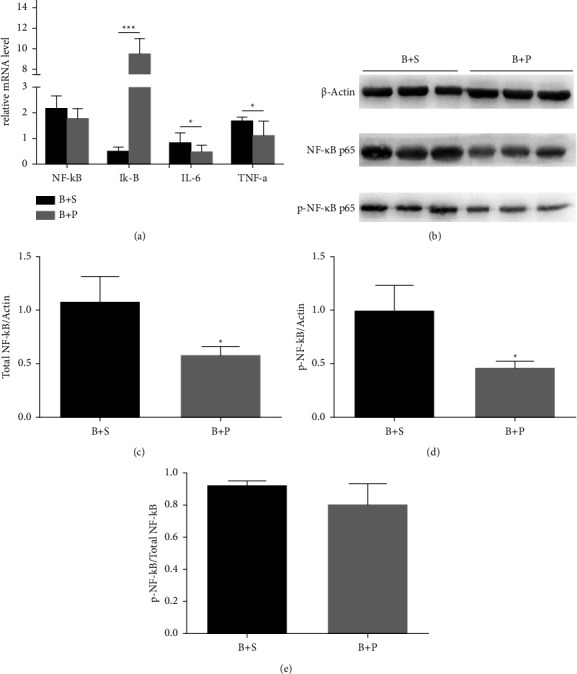
Effects of blue light on NF-*κ*B activation. (a) Real-time PCR for the expression of cytokines and inflammatory factors. The production of IL-6 and TNF decreased significantly after PDTC treatment (^*∗*^*P* < 0.05), while the expression of I*κ*-B upregulated significantly (^*∗∗∗*^*P* < 0.001). (b) Effects of blue light on the activation of NF-*κ*B in corneas are evaluated by Western blot analysis using *β*-actin as a loading control. (c, d) PDTC treatment decreased the expression of total NF-*κ*B p65 and phosphorylated NF-*κ*B p65 (^*∗*^*P* < 0.05). The ratio of phosphorylated NF-*κ*B p65 to total NF-*κ*B p65 showed no significant difference.

The results showed that TNF-*α* and IL-6 levels were significantly suppressed by PDTC treatment. The NF-*κ*B level in the PDTC group decreased slightly while the I-*κ*B was markedly increased (*P* < 0.001, Figure 7(a)). The data of Western blot showed that total NF-*κ*B p65 and pp65 significantly decreased after the PDTC treatment (*P* < 0.05, Figures 7(b)–7(d)). However, there was no significant difference in the ratio of phosphorylated NF-*κ*B p65 to total NF-*κ*B p65 between the two groups (Figure 7(e)).

## 4. Discussion

Under the background of an energy-saving policy, LED has gained strong growth because of its energy-saving performance. At the same time, we should pay particular attention to its biosecurity due to its unique technological characteristics [21]. Phototoxicity of visible light, especially blue light, has been widely studied [2, 21], but only minor attention has been paid to the ocular surface [22]. The short wave blue light between 415 and 455 nm is the most harmful because it can directly enter the retina through the lens and cause irreversible photochemical retinal damage, which is called blue light hazard [1]. For the mass production of white LEDs, blue diodes were the irreplaceable components, whose wavelength is between 440 and 475 nm [2]. Therefore, we chose 450 nm blue light to detect the potential hazard on the rat's ocular surface.

The cornea is located at the front of the eyeball and is the first structure encountered when light passes through the eye [23].High-energy blue light is close to ultraviolet in the color spectrum, so overexposure to blue light is also dangerous to the ocular surface. Researchers have indicated that the survival rate of corneal epithelial cells decreases after blue ray irradiation [24]. The oxidative damage caused by blue light was reduced by the effective antioxidant extract associated with free radical elimination, thus improving the clinical symptoms of the eye surface in a dry eye mouse model [20]. It further confirmed that blue light can influence the ocular surface through inflammation and oxidative stress, which may be related to the formation of dry eyes [7].

To further explore the underlining mechanism of blue light injury on the ocular surface, a special LED device that can emit pure blue light was used in our study. Blue light, which is approximately 20–60 fold more intense than the UV used in previous studies, may be hazardous to corneal epithelial cells undergoing mitosis [12]. This means that much more energy from blue light is required to damage the cornea than from UVB.

According to the results of the pilot experiment, the blue light with 1000 lux illumination was selected. Rats were irradiated with 126 J/cm^2^ each day for a consecutive 28-day period. The daily irradiation dose was similar to the previous experiment [7].

We found the changes in the ocular surface after 28-day blue light emission was in line with dry eye syndrome and manifested as tear film instability and corneal epithelial staining. As for TBUT, a significant decrease was observed in the blue light group, which indicates the disruption of tear film stability. These changes in clinical parameters are in line with dry eye models in previous studies [7, 25]. Although the shortening of TBUT and corneal staining appeared in the control group due to the environmental changes, the statistical difference between the groups at the same time point suggested that blue light treatment was the main factor of injury in the irradiation group. However, as shown by the phenol red line test, there was no significant change in the amount of tear secretion in the two groups. Unlike other modeling methods [25–27] (removal of the lacrimal gland, topical administration of preservatives, or injection of anisodamine), we considered that light might have less interference with the lacrimal gland and tear secretion, while ocular surface inflammation and other changes appeared despite no change in tear production [28].

To further explore the underlying causes of the cornea and the instability of the tear film, PAS staining was used to detect the distribution of goblet cells in the conjunctiva [29]. Mucin secreted by conjunctival epithelial cells and goblet cells (GCs) is also a key factor affecting the quality and stability of the tear film [30]. PAS staining indicated the number of conjunctival goblet cells in the blue light group was significantly reduced with morphological changes. It is well known that ocular surface inflammation can reduce conjunctival goblet cells [31], while IL-6 and IFN-*γ* could promote the loss of conjunctival goblet cells under desiccating stress [32, 33]. Therefore, we inferred that corneal chronic inflammation induced by blue light may disturb the ocular surface microenvironment and result in dry eye symptoms. However, Lee et al. reported that there was no significant difference in goblet cell density between UT and the blue light group [7]. This may be related to exposure time and animal species, so this factor requires further exploration.

Previous studies have shown that the damage mechanism of blue light on the ocular surface includes oxidative stress injury, ocular surface inflammation, and apoptosis [10]. Inflammation is a response to cell and tissue damage intended to accommodate changing extracellular stresses and stimuli [18], which is considered the key cause and result of dry eye [34]. In our present study, increased mRNA levels of NF-*κ*B, TNF-*α*, and IL-6 were observed in the blue light group compared to the control group. The inflammatory reaction produces TNF-*α*, IL-6, and other inflammatory factors, which induce goblet cell apoptosis and reduce mucus secretion, thus affecting the stability of the tear film [23, 35, 36]. Moreover, we observed the disruption of corneal structures after 28-day blue light exposure along with the upregulation of inflammatory factors. Furthermore, desmosome junctions also decreased under the electron microscope in the blue light group, which is similar to the microscopic appearance of dry eyes caused by particulate matter 2.5 (PM2.5) [37]. We speculated that the cytokines involved in the injury mechanisms of human corneal epithelial cells may lead to further damage to the cornea. This change may be a manifestation of the degeneration caused by the blue light exposure.

Dry eye is a common ocular surface inflammatory disease that significantly affects the quality of life [38]. Desiccating or osmotic stress to the ocular surface epithelium is sufficient to activate the mitogen-activated protein kinase (MAPK) and NF-*κ*B pathways [28, 39]. NF-*κ*B can regulate the gene expression of a variety of cytokines and adhesion molecules involved in the inflammatory response, which is closely related to the occurrence of inflammation [40, 41]. It is also a common downstream event in most epithelial stress responses, and increased NF-*κ*B signaling in the corneal and conjunctival epithelia has been reported in different models of ocular surface disorders [42, 43]. In our study, the enhanced expressions of IL-6 and TNF-*α* in the blue light group are considered key inflammatory factors in NF-*κ*B signaling pathway. We inferred that overexposure to blue light might cause cornea damage, which attracted macrophages and other cells to liberate proinflammatory mediators. In the previous study of dry eye caused by ocular surface stimulation, inflammation after exposure to proinflammatory cytokines mainly depended on the activation of NF-*κ*B. For example, TNF-*α* may induce multiple cellular effects through NF-*κ*B signaling pathways such as apoptosis [44], cell death, and production of chemokines [45]. In our study, the protein expressions of TNF-*α*, NF-*κ*B p65, and pNF-*κ*B p65 increased significantly after 28-day blue light irradiation. The increased ratio of pNF-*κ*B p65 to total NF-*κ*B p65 was also recorded, which was regarded as an indicator of NF-*κ*B activation [19, 37, 46]. Consequently, the activation of NF-*κ*B may be one mechanism of blue light-induced dry eye.

Excessive NF-*κ*B activation in epithelial cells promotes mucosal inflammation, while many reports have shown that N-acetyl-l-cysteine (NAC) and PDTC inhibit the activation of NF-*κ*B in different types of cells [47]. The reported mechanisms of PDTC in previous studies include restoring conjunctival tolerance in allergic conjunctivitis [16] and preventing dry stress-induced skewing in the mucosal immune response [17]. On account of its potent anti-inflammatory effects, we hypothesized that if light damage to the ocular surface is indeed based on NF-*κ*B pathway activation, its prevention by PDTC should slow disease development. The most obvious change is that the mRNA expression of the PDTC group showed an increased value of I-*κ*B. And the dry eye symptoms of rats in the inhibitor group were effectively alleviated. I*κ*B, as an inhibitor protein of NF-*κ*B, usually interacts with p65 in the cell cytoplasm. Once activated by pathological or physiological stimulation, I-*κ*B was phosphorylated and degraded [42]. PDTC could block the process downstream of I-*κ*Ba phosphorylation and upstream of I-*κ*B degradation. Our results showed that inhibition of NF-*κ*B signaling can reduce the level of inflammatory molecules. Regarding Western blot analysis, the protein levels of p65 and pp65 in the PDTC group were downregulated and significantly lower than those in the saline group, which is consistent with previous dry eye treatment models [25]. At the same time, samples in the PDTC group showed a thinner epithelium layer and more goblet cells, close to but below the normal level. Our results showed that blue light may cause cornea damage through NF-*κ*B activation, while PDTC might reverse it.

Limitations of the study include the use of light with a fixed wavelength and intensity. To explore the specific damage of blue light on the ocular surface, we chose 1000 lux blue light in our study, which could not mimic the daily lighting system. However, it may enhance awareness of blue light protection, especially for susceptible people such as the elderly, children, and people working in special environments. At last, the NF-*κ*B pathway is complicated, and as an important component in a chain of events leading to ocular mucosal tolerance to a topical surrogate antigen, further study on the details of activation is required. We believed that the data in our study would facilitate further research.

In conclusion, our results demonstrated a close relationship between blue light overexposure and dry eye syndrome. The mechanism may be related to inflammation and the activation of the NF-*κ*B pathway. The blue light hazard was related to the blue light intensity received by the eye, illumination distance, the spectrum of the light source, irradiation time, and so on. Although human beings are only exposed to high-energy intensity blue light for a long time in some specific working environments, the cumulative effect of blue light on the ocular surface should not be ignored [48, 49].

## 5. Conclusions

1000 lux LED blue light irradiation for 28 days can induce dry eye symptoms on the ocular surface of rats, including tear film instability and corneal epithelial damage, which may be related to the decrease of conjunctival goblet cells. Furthermore, the mechanism of blue light-induced ocular surface damage is related to inflammation. Blue light can induce the upregulation of NF-*κ*B, IL-6, and TNF-*α* expression in ocular surface tissues. The NF-*κ*B pathway may be involved in the pathogenesis and progression of ocular surface light damage.

## Data Availability

The data used to support the findings of this study are available from the corresponding author upon request.

## Abbreviations

LED: Light-emitting diode
TBUT: Tear breakup time
HE: Hematoxylin and eosin staining
PAS: Periodic acid–Schiff
qPCR: Quantitative real-time polymerase chain reaction
WB: Western blot
NF-*κ*B: Nuclear factor kappa B
I-*κ*B: Inhibitory kappa B
IL-6: Interlukin-6
TNF-*α*: Tumor necrosis factor-*α*
PVDF: Polyvinylidene fluoride
PDTC: Pyrrolidine dithiocarbamate
UT: Untreated group
BL: Blue light group.
